# Supplementary material for: EnzML: multi-label prediction of enzyme classes using InterPro signatures
Source: BMC Bioinformatics. 2012 Apr 25;13:61. doi: 10.1186/1471-2105-13-61 (PMC3483700; doi:10.1186/1471-2105-13-61)
Supplement: Addtional file 5 — The Java code to format the data files, evaluate and predict. The file enzml_java_code.tar.gz contains the Java code used to format database data to ARFF and XML formats, to execute cross and train-test (jackknife) evaluations and to record evaluation results to database. More information is included in the readme.txt file and the Javadoc files. The code can be used with a MySQL database. To use a different database software, other JDBC drivers might be required. [file 1471-2105-13-61-S5.gz › java_code/utils/doc/uk/ac/ed/inf/utils/guiutils/CommandLineMenu.html]

CommandLineMenu


---


|  |  |  |
| --- | --- | --- |
| **Overview** \| **Package** \| **Class** \| **Use** \| **Tree** \| **Deprecated** \| **Index** \| **Help** | |  |
| PREV CLASS   **NEXT CLASS** | **FRAMES**    **NO FRAMES**     **All Classes** |  |
| SUMMARY: NESTED \| FIELD \| CONSTR \| METHOD | DETAIL: FIELD \| CONSTR \| METHOD |  |


---


## uk.ac.ed.inf.utils.guiutils Class CommandLineMenu

```
java.lang.Object
  uk.ac.ed.inf.utils.guiutils.CommandLineMenu
```

---

``` public class CommandLineMenu extends java.lang.Object ```

Stores the options for a command line program.

**Version:**
:   14 Dec 2007

**Author:**
:   Luna De Ferrari luna.deferrari-at-ed.ac.uk

---

**Constructor Summary**

`CommandLineMenu(java.lang.String name,
java.lang.String programDescription,
java.util.ArrayList<CommandOption> options,
java.lang.String exampleCommand,
java.lang.String authors)`


| **Method Summary** | |
| --- | --- |
| `java.lang.String` | `toString()`             "-n  The file containing the network e.g. |

**Methods inherited from class java.lang.Object**

`equals, getClass, hashCode, notify, notifyAll, wait, wait, wait`

**Constructor Detail**

### CommandLineMenu

```
public CommandLineMenu(java.lang.String name,
                       java.lang.String programDescription,
                       java.util.ArrayList<CommandOption> options,
                       java.lang.String exampleCommand,
                       java.lang.String authors)
```


**Method Detail**

### toString

```
public java.lang.String toString()
```

:   "-n  The file containing the network e.g. in sif format (any Cytoscape format is valid http://www.cytoscape.org/)"
    + "\n" +"-o  File containing the node names to act as origin for the path search (one per line)"
    + "\n" +"-t  File containing the node names to act as targets for the path search (one per line)"
    + "\n" +
    "-r  The path and name of the report file to be created"
    + "\n" +"-s  e.g. \\s for space \\t for tab or , (comma). Default is tab separated."
    + "\n" +"-m method (algorithm) Either 0 = single shortest path (directed and undirected) or 1 = PESCA multi shortest path (all undirected shortest paths). Default is 0."
    + "\n" +"-a  (optional) The directory containing the .NA (node attributes) .EA (edge attributes) Cytoscape files."
    + "\n" + "-h > gets this help."+ "\n" +

    :   **Overrides:**: `toString` in class `java.lang.Object`


---


|  |  |  |
| --- | --- | --- |
| **Overview** \| **Package** \| **Class** \| **Use** \| **Tree** \| **Deprecated** \| **Index** \| **Help** | |  |
| PREV CLASS   **NEXT CLASS** | **FRAMES**    **NO FRAMES**     **All Classes** |  |
| SUMMARY: NESTED \| FIELD \| CONSTR \| METHOD | DETAIL: FIELD \| CONSTR \| METHOD |  |


---
